# Paper-Based Electroanalysis for Emerging Pollutant Detection

**DOI:** 10.1021/acselectrochem.5c00332

**Published:** 2025-11-04

**Authors:** Sima Singh, Alessandra Glovi, Gabriella Iula, Stefano Cinti

**Affiliations:** † Department of Pharmacy, 9307 University of Naples “Federico II”, Via D. Montesano 49, 80131 Naples, Italy; ‡ Scuola Superiore Meridionale, University of Naples “Federico II”, 80138 Naples, Italy; § Sbarro Institute for Cancer Research and Molecular Medicine, Center for Biotechnology, College of Science and Technology, Temple University, Philadelphia, Pennsylvania 19122, United States

**Keywords:** Emerging pollutants, paper-based electrochemical sensors (ePADs), sustainable sensing, environmental monitoring, microfluidics

## Abstract

Over the past two decades, the pollution lexicon has expanded beyond nutrients, hydrocarbons, and heavy metals to include emerging pollutants (EPs) and emerging contaminants (ECs), which now pose a critical challenge to environmental monitoring. Although, the conventional techniques are accurate and sensitive but often impractical for rapid, on-site monitoring. In response, a new wave of innovation has emerged to redefine environmental sensing through the development of paper-based electrochemical analytical devices (ePADs) which is a convergence of green chemistry, flexible electronics, and smart design. At the heart of their effectiveness lies in cellulose materials which depends on renewability, biodegradability, capillarity, and flexibility enable effective, low impact ePADs for passive fluid handling and microfluidics. Advances in 3D origami-based ePADs enable multi-analyte sensing, and, when paired with green manufacturing and smartphone-linked, low-power electronics, deliver real-time, cloud-ready data. Achieving widespread, sustainable deployment for decentralized pollution monitoring will require standardized validation, scalable manufacturing, and collaboration across scientific, technological, and policy domains. Looking forward, more than a replacement for conventional techniques, ePADs invite us to rethink our relationship with the environment. It signals a new contract between innovation and the planet-one in which analytical performance is inseparable from ecological responsibility. Each cellulose channel and fold demonstrates that high-precision sensing can be lightweight, biodegradable, and accessible. In this vision, smarter technology is also gentler, delivering cleaner water, healthier communities, and a more resilient Earth.

## Graphical abstract

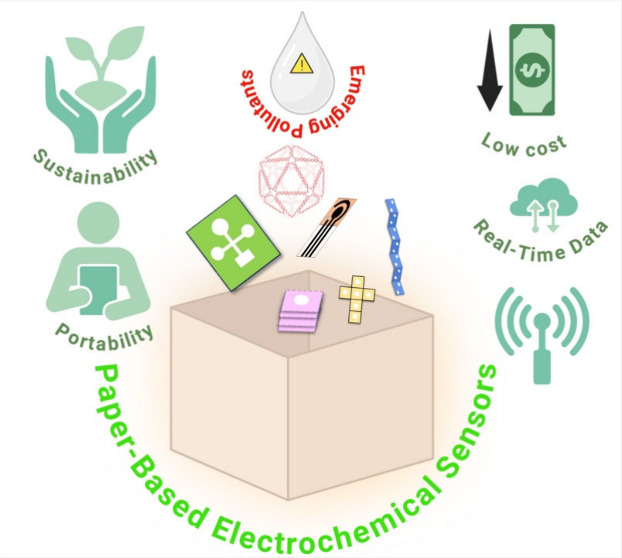

## Introduction

Over the past two decades, the lexicon of environmental pollution has expanded well beyond the classic trio of nutrients, hydrocarbons, and heavy metals. In this context, emerging pollutants (EPs) are organic or inorganic substances. They occur in environmental compartments at atypically high concentrations. These levels are often at or above guideline or background values. EPs include legacy chemicals present at elevated loads. They also include novel toxicants with demonstrated ecological and human health effects.^1–3^ Emerging contaminants (ECs) are newer synthetic chemicals lacking clear regulation. Even at trace ppt-ppb levels, they can pose outsized risks to ecosystems and human health, warranting regular monitoring.^4^

While the terms are frequently used interchangeably, EPs emphasize concentration exceedances with established harm, whereas ECs now encompass diverse classes, including trace metals associated with electronics miniaturization, widely used pesticide classes (e.g., organophosphates and carbamates), pharmaceutical residues (antibiotics, antidepressants, hormones), endocrine-disrupting compounds, and highly persistent “forever chemicals” such as per- and polyfluoroalkyl substances (PFAS).^5^

For example, PFAS have been detected in drinking water at concentrations up to hundreds of ng/L in contaminated regions.^6^ Pharmaceuticals and personal care product residues are frequently found in surface waters at low μg/L levels worldwide.^7^ Toxicological studies implicate many ECs at these concentrations in neurotoxicity, endocrine disruption, immunosuppression, and carcinogenesis.^8,9^ The U.S. Environmental Protection Agency (EPA) established advisory limits for drinking water which include a 4 ng L^–1^ threshold for perfluorooctanoic acid (PFOA) and they are reviewing exposure standards for related substances.^10^ Similar concerns extend to terrestrial systems. The pan-European LUCAS 2018 survey of agricultural soils revealed that 3.6% of sampled sites contained total pesticide residues above 0.5 mg kg^–1^, with a non-negligible subset exceeding 1 mg kg^–1^.^11,12^ These findings underscore the need for harmonized regulatory vigilance across environmental compartments. Given the proven mobility of many pesticides and their metabolites, such soil burdens raise the spectre of groundwater contamination and chronic dietary exposure, strengthening the case for ultra-trace monitoring tools that can operate across water, soil and food matrices.

The deployment of rapid, portable analytical platforms is becoming increasingly critical for the quantification of ECs in complex environmental matrices, particularly within resource-constrained settings lacking routine laboratory infrastructure.^13^ Definitive identification and quantitation at trace concentrations continue to rely on high-end instrumentation such as liquid chromatography coupled with mass spectrometry (LC-MS) and gas chromatography coupled with MS (GC-MS), which consistently achieve detection limits in the sub-nanogram per liter range.^14^ However, these methodologies necessitate cold-chain sample preservation, laborious solvent-based extraction protocols, and technically trained personnel, thereby limiting their applicability for high-resolution temporal and spatial wide-area monitoring. This practical gap has catalyzed the rise of sustainable, paper-based electrochemical sensors (PES), which integrate sample handling, analyte recognition and signal transduction on a single disposable cellulose platform. Building on this foundational shift, a key advantage of PES lies in their material and environmental sustainability, especially through the use of cellulose as a core substrate.^15–17^

Sustainability is not a marketing label but a core design constraint for these technologies. PES are increasingly viewed as that platform. Cellulose offers pump-free capillary flow, a porous, high-surface-area scaffold for reagent storage and pre-concentration, and a biodegradable substrate that can be incinerated in a single step. These structural and ecological advantages are not merely theoretical but they have enabled new levels of analytical performance across diverse contaminant classes.^18–21^ The detection of ethinylestradiol in river water reached 0.1 ng L^–1^ using Osteryoung square-wave voltammetry through a silica-nanoparticle-functionalized paper micro-zone integrated onto a reduced-graphene electrode.^22^ A reagent-free strip quantifies bisphenol A (BPA) on printed carbon tracks without auxiliary chemicals, simplifying the workflow while maintaining sub-μg L^–1^ sensitivity.^23^ Similarly, research findings show that biodegradable paper platforms improve analytical sensitivity while producing significantly less plastic waste than conventional polymer substrates.^24^ In tandem with material innovation, architectural advancements in device design have expanded functionality. Foldable “origami” sheets integrate multiple enzyme-based sensing zones for multiplex pesticide analysis without external pumps^25,26^ and a three-dimensional potentiometric platform couples a solid-contact reference electrode to on-board bioreceptors, enabling USB-powered detection of enzyme inhibition by pesticides in a palm-sized device.^27^

Importantly, even the analytically elusive PFAS family has been brought into reach. PFAS are persistent pollutants with health risks, requiring better detection tools. This study reports an organic electrochemical transistor (OECT) sensor with a molecularly imprinted polyaniline (PANI) gate that selectively detects PFOA in seawater. The sensor shows strong specificity for PFOA over other PFAS and surfactants, with a detection limit of 1.6 ppt-well below EPA guidelines. This demonstrates a low-cost, rapid, and selective method for monitoring PFOA in complex environments.^28^

Yet despite these promising results, a critical frontier remains: field validation on unspiked environmental samples is still pending. To further democratise use, recent designs increasingly prioritise portability and user accessibility through digital integration. This shift is illustrated by hybrid devices that integrate electronic coupled with smartphone apps for real-time, user-friendly interpretation. A nanopaper “smart device” couples electrochemical and optical signals from the same paraoxon inhibition reaction, with data captured by an android application linked to a miniature potentiostat.^29^ Smartphone-connected and artificial intelligence (AI)-assisted readers can streamline result interpretation, automate calibration, and enhance decision-making accuracy in decentralized settings.^30^ Such digital interfaces lower the barrier for non-specialists to generate defensible data at the point-of-need. These advances highlight the transformative potential of combining biodegradable substrates with low-power, smart electronics for autonomous, field-ready environmental diagnostics. This synergy is redefining the scope of environmental monitoring technologies.

However, the real-world utility of PES depends on their ability to deliver precise, reproducible, and durable performance in diverse and resource-limited settings.^31^ As environmental contaminants spread across increasingly complex ecosystems, the need extends beyond detection to sensing platforms that are affordable, user-friendly, and ecologically sustainable. PES meet these demands through low-cost fabrication from renewable materials, intuitive and power-efficient operation, and modular adaptability across various analytes and matrices. With their growing integration into digital ecosystems and grounding in green chemistry, PES are evolving into scalable solutions for environmental justice, regulatory compliance, and sustainable public health surveillance. This review summarizes the growing occurrence of ECs and the limitations of conventional laboratory assays for field use. It evaluates the design, analytical performance, and sustainability of PES as green tools for decentralized EC monitoring. Recent origami-folded and laminated architectures convert planar paper into compact lab-on-paper systems with three-dimensional flow and multilayer integration. Paper serves as both substrate and microfluidic network, enabling capillary-driven transport without pumps, while integrated electrodes provide sensitive detection of diverse pollutants. With smartphone-based data acquisition and on-device analytics, these platforms are well suited for in situ environmental monitoring (Figure 1).

**Figure 1:**
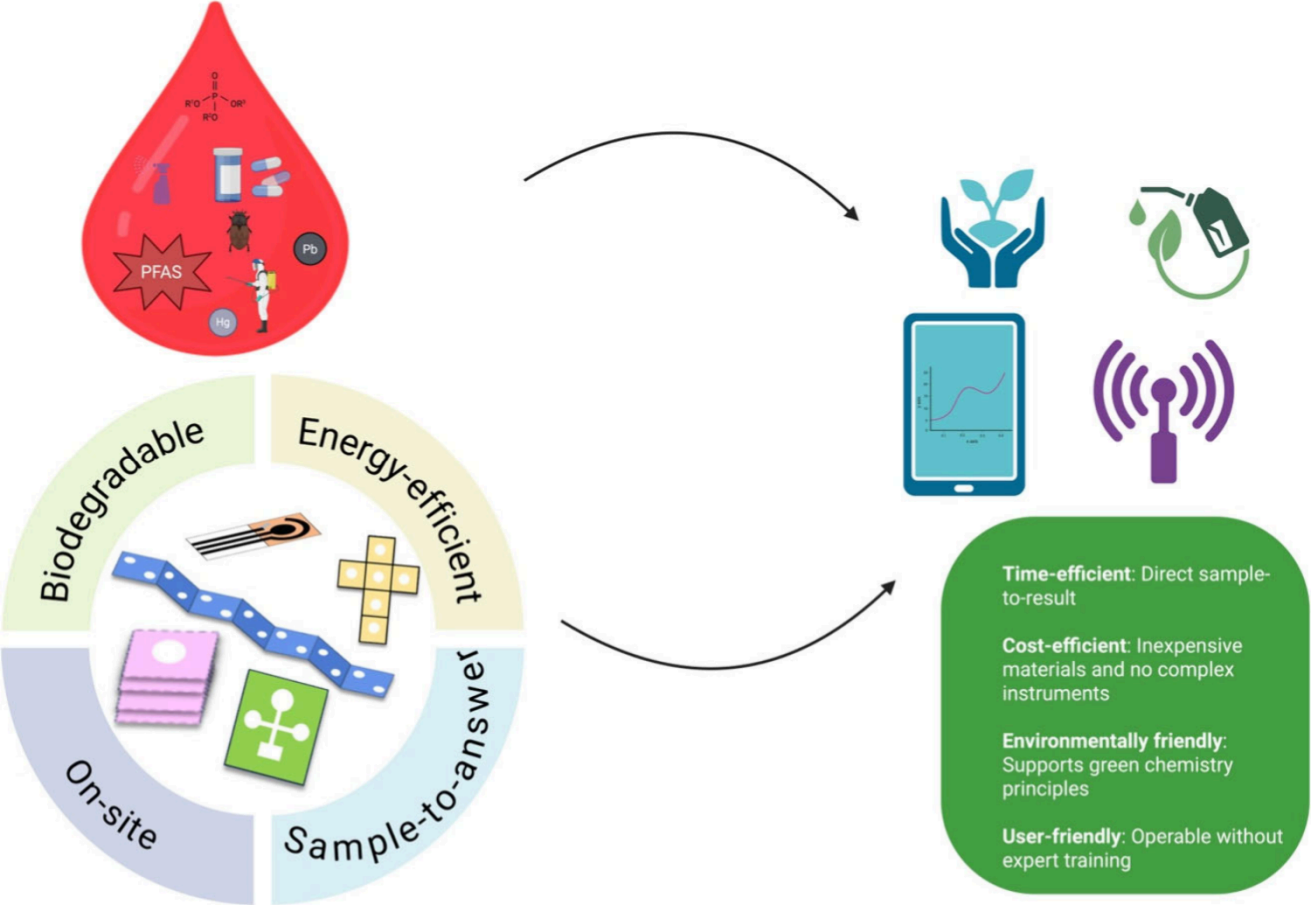
Schematic representation of the fabrication process for PES. This approach integrates multiple patterned layers into a single biodegradable device, enabling on-site, sample-to-answer, and energy-efficient sensing.

## Limitations of Conventional Analytical Workflows for Emerging Contaminants

Environmental monitoring of EC, including pharmaceuticals, pesticides, and industrial additives, traditionally uses centralized laboratory workflows with discrete sampling, pre-concentration techniques, and GC-MS analysis.^32–34^ These techniques, while providing high sensitivity and specificity for detecting ECs in water, are resource-intensive, time-consuming, and require substantial infrastructure and expert personnel.^35,36^ Despite being the regulatory “gold standard”, these methods have limitations, including lengthy sample preparation, analysis times, and limited spatial and temporal coverage. Figure 2 shows key limitations of centralized traditional lab for emerging contaminant detection. Despite high analytical precision, centralized systems are hindered by time, cost, sustainability, and limited accessibility in resource-constrained areas. These gaps underscore the need for portable, low-power solutions like PES.s

**Figure 2:**
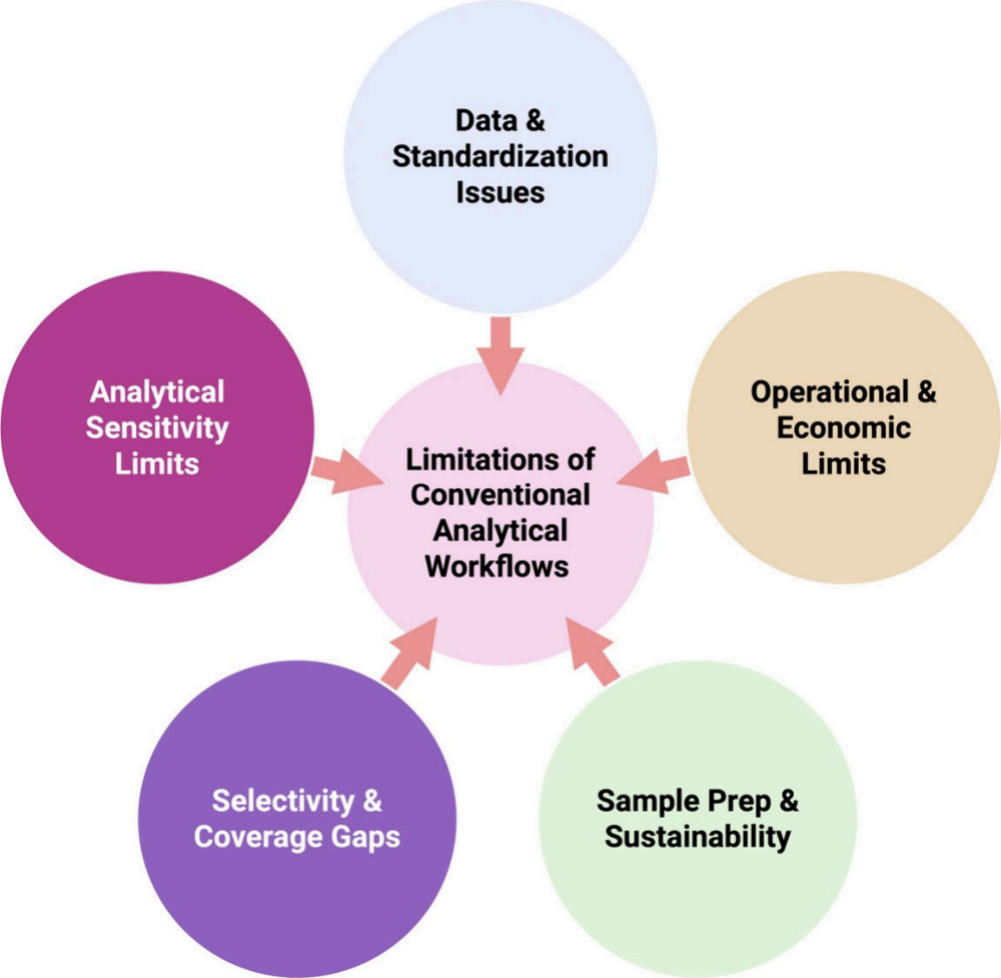
Current analytical methods for detecting emerging contaminants face multiple critical restrictions in their conventional centralized operation. The equipment-based system requires extensive sample preparation, transport time, expensive, environmentally damaging through solvent usage and energy consumption. The high sensitivity of these methods does not compensate for the limitations which prevent their use in real-time and field applications.

The paragraphs below outline the principal limitations that motivate the search for portable, electrochemical (and other fieldable) sensing alternatives.

### Analytical sensitivity limits in real-world matrices

Matrix components such as surfactants, humic colloids, and salts suppress electrospray ionization by more than 50%. As a result, liter-scale sampling and extensive clean-up procedures are necessary to achieve sub-ppt detection targets.^37,38^ The new U.S. drinking water limits, set at less than 4 ng/L for PFOS and PFOA.^39^ It often require isotope dilution, dual-column setups, and repeated injections. However, issues like carry-over and tailing of PFOA and PFOS still increase the quantification limits.^40^

### Selectivity and Chemical-Coverage Gaps

Selectivity and compound coverage are equally problematic. EPA methods 533/537 enumerate fewer than 30 PFAS out of the >3000 precursors now known, while thousands of pharmaceuticals, pesticides, and transformation products remain invisible to triple-quadrupole screening.^41^ For >80% of suspects detected during non-target High-Resolution Mass Spectrometry (HRMS) interrogations no authentic or isotopically-labelled standards exist, preventing “Level 1” identification and thwarting enforcement or risk assessment.^42^ Competing surfactants further distort solid-phase extraction (SPE) recoveries and chromatographic resolution, undermining both qualitative and quantitative confidence.^43,44^

### Sample-Preparation Burden and Sustainability Concerns

The SPE-LC-MS process generates large volumes of waste because the precondition and eluate steps require tens to hundreds of milliliters of solvent for each large-volume sample.^45^ The daily power consumption of ultra-high performance liquid chromatography-mass spectrometry (UHPLC-MS) instruments under standard laboratory operation amounts to 4.5–5.0 kWh according to Agilent, Shimadzu and Thermo specifications but can increase to 6.7 kWh per day.^46^ The use of centralized labs results in delayed shipments and queue times which extend the process by several days to weeks while producing additional emissions.^47,48^ The use of fluoropolymer labware in PFAS applications leads to secondary contamination that requires additional blank and rinse procedures.^44,49^

### Operational and Economic Constraints

High-performance LC-MS/MS systems typically require significant capital investments, often exceeding USD $10,000-$500,000, depending on system configuration and resolution.^50^ The operation of these systems requires climate-controlled laboratory facilities and continuous high-vacuum pumping systems and trained personnel which most small utility organizations and field agencies lack the ability to maintain.^48^ Remote samples need to be chilled before shipping which creates a time delay of several days to weeks while substances in the samples can transform and the ability to track changes over time becomes restricted because grab samples fail to detect peak storm-water/WWTP levels and autosampler-HRMS operations generate massive data sets that need external processing to prevent real-time monitoring.^51^

### Data-Handling and Standardization Challenges

The untargeted analysis of environmental samples through HRMS generates numerous features which require expert verification to eliminate false positive results. The use of different peak-picking algorithms in non-target LC-HRMS workflows creates challenges for achieving reproducible results.^52^ PFAS monitoring faces a major challenge because it lacks sufficient standardized data that tracks PFAS levels over time (e.g., single time-point serum studies).^53^ The added power of instruments becomes weakly effective for generating actionable insights when standardized quality assurance and quality control (QA/QC) and reporting systems are not standardized. Real-time sensors that provide calibrated matrix-corrected concentration data would reduce the need for extensive informatics processing.

This persistent gap between regulatory demands and what centralized LC/GC-MS workflows can realistically deliver, creates a clear rationale for shifting the transduction step to electrochemistry as the core sensing mechanism. Electrochemical detection is inherently compact and energy-efficient; it generates a quantitative signal directly from charge-transfer events.^54,55^ When paired with paper-based substrates, the approach becomes even more compelling: paper is cheap, lightweight, biodegradable, and compatible with scalable printing techniques.^56^ It also enables passive fluid transport via capillary action, removing the need for pumps or valves and reducing the total system footprint.^20^ This wicking phenomenon is further modeled by Darcy’s and Washburn’s laws, highlighting how paper porosity, pore size, and surface wettability control fluid flow autonomously.^57^ The essential analytical metrics between conventional methods and ePADs are compared in Table 1.

**Table 1:** Key Performance Difference between Conventional and ePADs

Parameter	Conventional analytical methods	ePADs
Equipment	Require large, expensive instruments like Atomic Absorption Spectroscopy (AAS), Inductively Coupled Plasma (ICP), HPLC, Nuclear Magnetic Resonance (NMR), etc.	Paper substrates with printed/formed electrodes; integrated modifiers/biorecognition; handheld/smartphone readers demonstrated.^58,59^
Portability	Non-portable; lab-bound.	Fully portable; paper substrates with printed electrodes; fieldable electronics and smartphone interfaces demonstrated. Examples: office-paper strip used on-site for soils/produce;^24^ Arduino-based portable EIS reader;^30^ smartphone-operated nano-PAD;^29^ light-addressable PEC array with single-channel analyzer.^60^
Detection limit	Ultra-trace, often <1 ng/L (ppt-low ppb), method-dependent across platforms.^61^	Typically ng/L-μg/L (ppb–ppm), depending on recognition layer and transduction; examples: chlorpyrifos 3 ng/L (EIS, 100 μL);^30^ PFOS ≈1 ppt with MIP-polyaniline (resistive readout);^62^ OPs in soil extracts ∼1.3 ng/mL on office-paper strip.^24^
Time Efficiency	Multistep prep; transport/queueing prolong total turnaround.	Rapid readout with minimal prep; seconds-minutes on device; high-throughput PEC addressing.^63^
Selectivity/specificity	Excellent chemical resolution and confirmation via mass spectral/atomic signatures; gold standard for specificity.^64^	Biorecognition or synthetic affinity layers; cross-reactivity must be managed.^65^
Cost	High capital and operational cost.^66^	Low-cost, disposable paper formats; sustainable substrates (office paper, nanocellulose).^67^
Sample preparation	Often multi-step	Minimal prep; on-paper handling.^68^
Sample volume	Often milliliters after prep/extraction.	Microliter-scale on paper; examples: 100 μL;^30^ 30 μL droplet.^62^
Environmental Impact	Uses significant solvents/reagents; generates lab waste.	Minimal reagents; disposable paper; emphasis on sustainable materials.
Applications	Lab based	Point-of-need

## Overview: Fabrication Methods for PES

Microfluidic paper-based analytical devices (μPAD) offer a uniquely sustainable, low-cost, and scalable approach to environmental sensing. Their fabrication processes combine fluidic patterning, electrode integration, and functional layer deposition. All parameters are optimized for simplicity and mass production. The following subsections describe both fundamental and new fabrication techniques which have driven the development of PES.

One of the first steps in μPAD fabrication is defining the fluidic architecture. The most common method of wax printing involves applying solid wax which melts briefly to penetrate cellulose fibers and create hydrophobic barriers that restrict fluid movement. The method provides low-cost simplicity yet its diffusion process restricts the achievable resolution. The heat-transfer method relies on desktop printers and solid-ink plotters to transfer wax through office paper within less than 1 min.^69,70^ Wax printing also underpins inexpensive three-electrode cells on office copy paper, driving a 96% material-cost reduction compared with chromatographic substrates.^71^ Wax printing followed by thermal re-flow remains the most widely adopted patterning method because it combines low cost with moderate resolution; nevertheless, photolithography with UV-curable resists and CO_2_/UV laser ablation are increasingly preferred or solvent resistance are required.^65,72^ Folding or lamination (“origami”) strategies allow multiple patterned layers to be stacked or aligned, enabling vertical flow paths and compact three-electrode cells without enlarging the lateral footprint of the device.^65,73^

Electrode integration is another core component, as the electrochemical interface determines the sensor’s responsiveness and accuracy. Screen-printing conductive inks represents the primary method for creating electrodes. The three most frequently used inks for this purpose are carbon, silver/silver chloride and gold. Mass production of reproducible and cost-effective working, reference, and counter electrodes is possible through this method. For electrode formation, screen printing of carbon or Ag/AgCl inks still dominates owing to its simplicity and ability to deposit all three electrodes in a single pass.^74^ These printed electrodes are compatible with most pollutant classes, from heavy metals to pharmaceuticals. A major milestone after 2021 is the advent of laser-induced pyrolysis of cellulose: a single pulsed-laser scan converts the paper itself into porous graphitic tracks that retain wicking ability, eliminating the need for extrinsic inks while supporting high electroactive surface area. This “laser-pyrolyzed electrofluidics” approach can yield hundreds of sensors per sheet at a cost below $0.02 each and is now discussed in most forward-looking reviews as a scalable alternative to printing.^75,76^

The assembly of devices through lamination with pressure-sensitive adhesives, crease-programmed folding, and vertical stacking. The methods combine fluidic paths with embedded electrodes and reservoirs and connectors. The designs improve sample handling capabilities and support multiplexed assays and store reagents onboard while maintaining capillary flow.^77,78^ Reviews dedicated to biomedical and immunosensing applications emphasize how electrode orientation, substrate thickness, and barrier depth collectively influence signal stability and limit-of-detection in complex matrices.^65^ Finally, functionalization and surface modifications of electrodes enhance selectivity and sensitivity. Techniques include drop-casting nanomaterials, electrodeposition, or blending inks with recognition elements such as aptamers, molecularly imprinted polymers, and enzymes.^79^ These smart coatings can capture ultra-trace contaminants with high specificity.

Scalability and quality control receive growing attention. Roll-to-roll printing combined with in-line optical or electrical inspection is proposed to keep sheet-to-sheet variation in electrode resistance and to automate correction of barrier defects during production.^74,80^ Cleanroom-free prototyping techniques such as xurography and desktop laser cutting are also surveyed as gateways for laboratories in low-resource settings to iterate designs before committing to high-throughput manufacturing.^74,81^

Across the literature, authors concur that quantitative, side-by-side benchmarking of fabrication methods remains limited. Most studies offer qualitative or application-specific comparisons, lacking standardized metrics that would enable fair evaluation across materials and techniques. At the same time, sustainability has emerged as a central theme, with growing attention to biodegradable substrates, solvent-free processes, energy-efficient fabrication, and green ink formulations. These considerations increasingly appear alongside traditional performance metrics such as sensitivity and resolution, reflecting the broader push for eco-conscious sensor development. Looking ahead, key priorities include the use of uniform, reproducible paper substrates, cost-per-sensor analyses at the pilot production scale, and the validation of newer fabrication approaches such as electroless metal plating for stable reference electrodes and hybrid electrode assemblies compatible with Internet of Things (IoT)-integrated diagnostics. These directions highlight the field’s movement toward not only more capable sensors, but ones that are scalable, affordable, and environmentally responsible.

## Signal Transduction Mechanisms in PES

The electrochemical signals from chemical reactions at printed porous electrodes of PES produce electrical output. The selection of transduction methods depends on the electrochemical behavior of target substances and the operational characteristics of paper-based sensors including fluid movement and drying and minimal power requirements for handheld devices. The working electrode receives a time-dependent potential signal which generates faradaic current measurements from redox reactions at the interface. The stripping voltammetry method combines preconcentration of analytes on electrodes with subsequent potential sweep analysis to produce highly sensitive results through peak formation. The paper-based technique proves most beneficial for detecting trace heavy metals (e.g., Pb^2+^, Cd^2+^, Hg^2+^). It enables detection at parts-per-billion levels through origami μPADs which combine filtration with on-paper preconcentration. The detection method requires electroactive targets but faces challenges from deposition parameter sensitivity and electrolyte composition effects and it remains susceptible to matrix interferences and ohmic losses (iR drop) within the porous substrate.^63,82^

The amperometric method measures potential while tracking current changes over time through enzymatic product generation or mediator-based redox reactions. The technique provides several advantages because it operates at low power while accepting preloaded reagents and works at low potentials with Prussian Blue and suits cholinesterase-inhibition assays for detecting organophosphorus pesticides in soils and produce. The method faces three main drawbacks which include capacitive background signals, surface fouling and hydration-dependent signal instabilities that reduce measurement stability.^24,83^ Potentiometry determines the open-circuit potential difference between the indicator and reference electrodes through an ion-selective or molecularly imprinted membrane at near-zero current levels. The combination of paper substrates with solid-contact technology enables the development of inexpensive disposable devices that require minimal power to detect small organic pollutants through selective recognition mechanisms particularly useful for detecting endocrine disruptors like BPA with molecularly imprinted receptors. The method faces three main challenges which include potential drift, membrane-based selectivity restrictions and water-layer formation that can be addressed through suitable solid contact selection.^84,85^

Electrochemical impedance spectroscopy (EIS)­uses small AC perturbations to measure frequency-dependent impedance which enables tracking of charge-transfer resistance, double-layer capacitance and diffusion changes during binding or inhibition events. The technique provides label-free detection with ultra-low current measurements that detect interfacial chemistry changes effectively for weakly electroactive targets and enzyme/affinity assays such as AChE sensing of organophosphates. The method requires precise equivalent-circuit modeling yet paper porosity and hydration effects lead to reference-electrode instability and dispersion issues. The impedimetric/resistive measurement approach matches well with selective affinity layers for detecting electro-inert PFAS compounds.^30,86^

The combination of photoelectrochemistry (PEC) and electrogenerated chemiluminescence (ECL) on paper substrates provides three key advantages: ultra-low background signals, low power requirements and simple multiplexing capabilities for detecting electrochemically inert substances such as PFAS, pharmaceuticals/EDCs and some pesticides. The photocurrent in PEC systems becomes target-dependent when illuminated because the electrical readout operates independently from optical excitation which reduces matrix noise and allows low-bias detection. The ECL method provides light-based detection that minimizes electrical interference while allowing easy multiplexing through different emitter selection.^87^ Recent PEC work has demonstrated on-paper sensitivity and throughput compatible with field screening of small-molecule pollutants. A light-addressable paper PEC device patterned Ag nanowire/C60-Congo red photoanodes into 42 isolated zones and used acetylcholinesterase/thiocholine chemistry to quantify organophosphorus pesticides, achieving a 0.35 nM detection limit for DDVP over a 1 nM-22.5 μM range, with single-channel readout by moving the light spot zone-to-zone. This plate-like format illustrates how PEC on paper can deliver low limit of detection (LOD) and scalable multiplexing for on-site pollutant surveys.^60^ In ECL, electrochemical steps create excited states that emit photons detectable with simple cameras, yielding near-zero optical background and camera-based analyzers; early paper ECL showed phone imaging, and newer implementations are self-powered or ultra-low power.^88^

The PES system operates through five different transduction methods which include voltammetry, amperometry, potentiometry, EIS and PEC/ECL that work best with particular substances and operational conditions. The development of origami μPADs, solid contact materials and light-addressable PEC/ECL systems continues to advance PES technology for low-cost portable on-site pollutant detection despite its challenges with fouling and drift and matrix effects.

## Paper-Based Electrochemical Sensors for Emerging Pollutants

Growing evidence links trace-level anthropogenic contaminants to ecological harm, endocrine disruption and antibiotic-resistant pathogens. Laboratory chromatography provides accuracy but lacks portability and affordability. Paper-based electrochemical analytical devices integrate capillary-driven microfluidics with screen-printed three-electrode systems, enabling disposable, on-site monitoring with sensitivities reaching ng L^–1^ to pg L^–1^, while requiring only microliter-scale sample volumes and low-power handheld potentiostats. The review examines experimental studies from the last few years based on three essential requirements: (i) fabrication directly on cellulose, (ii) reporting of quantitative performance metrics such as LOD, selectivity, and, where available, response or analysis time, and (iii) the validation of sensor performance in buffer systems, preferably extended to real environmental matrices. The discussion covers a range of pollutant families, including pesticides, pharmaceutical residues, EDCs, PFAS, and industrial or heavy-metal micropollutants

### Pesticides: Organophosphates and Carbamates

Paper micro-devices have become one of the most active fronts in the fight against organophosphate (OP) and carbamate (CB) pesticides and persistent contaminants that increasingly fall under the umbrella term emerging pollutants. Their integration of enzyme-based bio-recognition with nanostructured materials on biodegradable substrates makes them suitable for field deployment, particularly in resource-limited settings. A notable example is the use of recycled office paper printed with Prussian blue-carbon black hybrids, which enabled butyrylcholinesterase (BChE)-based sensing of OPs in soil and produce with a detection limit of 1.3 ng mL^–1^ (Figure 3A).^24^ This underscores the synergy between eco-design and regulatory-grade performance. Advances in microfluidic structuring and dual-mode detection have enhanced both sensitivity and versatility.

**Figure 3:**
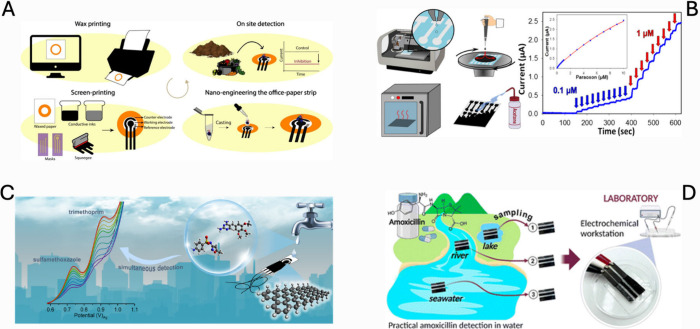
(A) Office paper-based electrochemical sensor: fabrication process and pesticide detection mechanism. Reproduced with permission from ref 24. Copyright 2021 American Chemical Society. (B) Schematic of graphene biosensor fabrication by inkjet maskless lithography and its application in organophosphate detection. Reproduced with permission from ref 89. Copyright 2018 American Chemical Society. (C) Simultaneous sensing of sulfamethoxazole and trimethoprim in water using a graphene nanoribbon-modified paper-based electrochemical device. Reproduced with permission from ref 90. Copyright 2021 Elsevier. (D) Sustainable cellulose nanofiber-based electrochemical platform for practical detection of amoxicillin. Reproduced with permission from ref 91. Copyright 2023 Royal Society of Chemistry.

Sharifi and colleagues developed nanocellulose-based paper devices incorporating bacterial and TEMPO-oxidised fibers, which produced simultaneous electrochemical and colorimetric signals without the need for external reagents, ideal for on-field paraoxon detection when paired with smartphone-readout systems.^29^ Such platforms demonstrate how sustainable materials can support multiplexed sensing and digital connectivity. Further innovations include the use of metal-organic frameworks (MOFs) for enzyme stabilization. A Zn-imidazolate MOF was used to immobilize AChE on a screen-printed micro-paper device, achieving rapid 5-second response times and a detection limit of 3 ng L^–1^ for chlorpyrifos via EIS, with additional integration of Arduino-based AI processing for intelligent data analysis.^30^ It illustrate a path toward intelligent field-deployed sensors for food-chain surveillance. Beyond inhibition assays, catalytic sensing strategies are gaining attention. One system featured laser-micropatterned, inkjet-printed graphene electrodes decorated with Pt nanoparticles for phosphotriesterase-mediated paraoxon hydrolysis, detecting p-nitrophenol at 3 nM within seconds (Figure 3B). It highlights the potential of non-inhibitory, direct transformation-based detection routes.^89^ Three-dimensional nanostructures have also expanded electrode design flexibility. ZnO nanorods and nanoplates deposited on porous carbon paper and cloth significantly improved AChE immobilization and signal output. In one study, the cloth-supported platform achieved a good LOD for paraoxon and successfully detected residues in apple juice and contaminated water, proving the robustness of textile-like “paper” architectures.^92^

Overall, PES have redefined how pesticide contamination is detected, shifting the focus from centralized laboratory diagnostics to portable, on-site analysis. Their adaptability, low production cost, and compatibility with eco-friendly materials make them particularly well-suited for widespread use in agricultural and environmental monitoring. By enabling rapid, user-friendly operation with minimal infrastructure, these platforms support a more accessible and sustainable approach to addressing chemical safety challenges in diverse settings.

### Pharmaceutical Residues

PES are increasingly being used to detect pharmaceutical residues in environmental and food samples. By combining cellulose-based materials with printed electrodes, these devices enable simple, pump-free fluid handling and single-use operation. Their low cost, portability, and compatibility with on-site testing make them well-suited for rapid screening of contaminants like antibiotics, even in complex sample matrices.

Antibiotic sensing dominates the recent literature. Martins et al. printed reduced graphene nanoribbon electrodes directly on cellulose to simultaneously quantify sulfamethoxazole (SMX) and trimethoprim (TMP) in surface water, demonstrating the first dual-analyte ePAD for these co-prescribed drugs (Figure 3C).^90^ A “green” approach was taken by Sari et al., who fabricated fully biodegradable cellulose-nanofibre electrodes decorated with polybenzimidazole-wrapped multi-walled carbon nanotubes; the device achieved a 0.3 μM detection limit for amoxicillin and functioned by simple soaking in tap and seawater samples (Figure 3D).^91^ Moving to biological matrices, Torrinha et al. showed that an unmodified carbon-fibre paper electrode could withstand fish-tissue homogenates after QuEChERS extraction, giving 0.065 μM LOD and 106% recoveries for trimethoprim.^93^ For meat safety, Deng and Yang printed a porous silver-nanoparticle/cellulose hydrogel onto paper strips, reaching 0.04 μM LOD for sulfamethoxazole with 86–92% recoveries in spiked pork homogenate.^94^ Collectively, these studies confirm that nanostructured carbon or metallic modifiers alone can deliver sub-μM sensitivity across water and food matrices without the need for molecular-recognition layers.

However, progress on non-antibiotic pharmaceuticals such as analgesics and antidepressants has been more limited. A notable exception is the work by Silva et al., who fabricated ePADs using a 3D-printed PLA stencil and homemade carbon ink. Their device successfully detected the antidepressant escitalopram at a limit of 0.5 μM in buffered solution demonstrating the viability of applying low-cost paper-based systems to broader pharmaceutical classes beyond antibiotics.^95^ Building on this trend, a recent innovation addresses the environmental detection of paracetamol, a widely used over-the-counter analgesic and antipyretic. Since 2020, paracetamol and other human pharmaceuticals have been classified by UNESCO as emerging contaminants and integrated into the UN’s 2030 Sustainable Development Goals. In response to its frequent detection in European water systems due to improper disposal, researchers developed a low-cost, all-in-one 2D paper-based electrochemical device. This platform integrates wax-printed fluidic channels and screen-printed electrodes on filter paper, allowing for rapid detection of paracetamol-within seconds and with a detection limit of ∼1 μM-without requiring complex sample pretreatment.^96^ This device not only simplifies environmental testing but also embodies a sustainable, scalable solution to monitor pharmaceuticals in wastewater, aligning effectively with global policy goals for clean water and responsible consumption.

Together, these examples illustrate the expanding utility of PES for pharmaceutical residue detection across a spectrum of analytes and sample types. While antibiotics remain the most extensively studied, recent advances signal growing attention to other drug classes, particularly through sustainable and decentralized sensing platforms.

### Endocrine-Disrupting Compounds

These advantages have catalyzed the development of increasingly sensitive and selective paper-based sensors for various EDCs, including BPA, phthalates, and steroid hormones. Early demonstrations concentrated on steroidal hormones. A landmark folding “origami” aptasensor for 17β-estradiol integrated filter holes, microfluidic channels, and a three-electrode system on a single sheet; a nanocomposite of amine-functionalized SWCNTs, new methylene blue, and AuNPs lowered the LOD to 5 pg mL^–1^ while allowing direct assays in clinical serum.^97^ Antibody-based recognition soon followed: a cellulose-microzone immunocapture assay pre-concentrated ethinylestradiol from river water, then transferred the zone to a graphene-modified screen-printed electrode for Osteryoung square-wave voltammetry, achieving an impressive 0.1 ng L^–1^ LOD and 40 min total assay time (Figure 4A).^22^

**Figure 4:**
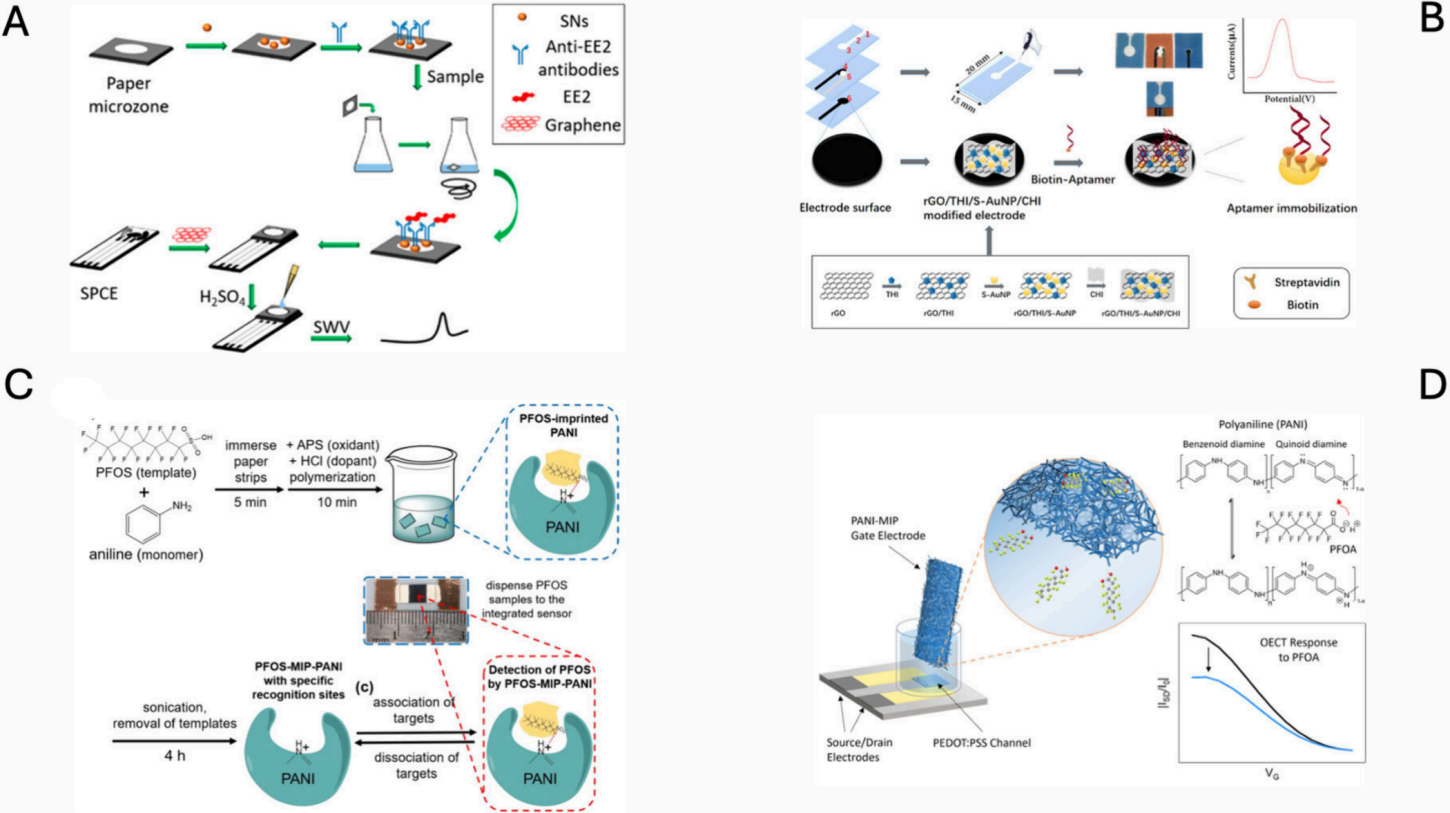
(A) Schematic representation of an electrochemical paper-based immunocapture assay (EPIA) for quantitative detection of Ethinylestradiol (EE2) in water samples. Reproduced with permission from ref 22. Copyright 2018 American Chemical Society. (B) Overview of nanomaterial fabrication, photographic evidence of μPEP construction, and standard response profile. Reproduced with permission from ref 98. Copyright 2021 American Chemical Society. (C) The detection of PFOS through electrochemical methods on paper substrates uses a molecularly imprinted polyaniline layer as the detection material. Reproduced with permission from ref 62. Copyright 2020 MDPI. (D) The OECT device with a PANI molecularly imprinted polymer as the gate electrode detects PFOA in seawater through selective detection. Reproduced with permission from ref 28. Copyright 2025 American Chemical Society.

More recently, biotin–streptavidin immobilisation of aptamers on a paper microfluidic chip has offered a versatile, label-free platform that was again validated with 17β-estradiol as the model analyte. The experimental results showed that this device allows the determination of 17β-E2 in a wide linear range of concentrations of 10 pg mL^–1^ to 100 ng mL^–1^ and the LOD is 10 pg mL^–1^ (Figure 4B).^98^

BPA represent another intensively studied EDC family. A reagent-free voltammetric strip fabricated by wax-patterned screen printing reached nanomolar detection levels for BPA in 2020, emphasizing ruggedness and field usability. A carbon black-modified paper sensor enabled BPA detection down to 0.03 μM, with strong linearity and recoveries in real water samples, demonstrating its low-cost, high-performance potential for on-site environmental monitoring.^23^ Molecularly imprinted nanobeads embedded in a disposable potentiometric paper strip subsequently provided a 0.15 μM LOD and successful quantification of BPA leached from commercial plastics, underscoring how ultra-low-cost substrates can host synthetic recognition layers without sacrificing selectivity.^85^ Community-oriented work has further demonstrated carbon-paste paper electrodes capable of detecting multiple bisphenol congeners down to 0.1 mM, a range suitable for gross contamination screening in resource-limited settings.^99^ While phthalate sensing on paper remains comparatively rare, a 2023 study introduced a buckypaper-integrated, screen-printed device in which the CNT mat serves as the working electrode; electrocatalytic oxidation of phthalates at -0.25 V could be read by a handheld potentiostat, illustrating both the chemical compatibility of CNTs with the aromatic ester analytes and the practical portability of the platform. The device provided a linear range of 70-15 ppm with a detection limit of 12.64 ppm and quantification limit as 42.03 ppm.^100^

In summary, cellulose-based electrochemical platforms have evolved from proof-of-concept hormone assays to sophisticated, nanomaterial-enhanced devices capable of ppt detection in real samples. Continued progress will likely hinge on integrating on-paper sample pre-treatment, expanding recognition chemistries to neglected EDC classes, and scaling multiplexed architectures-all while retaining the low-cost, disposable virtues that make paper uniquely suited to monitoring emerging pollutants.

### Forever Chemicals

PFAS pose a formidable challenge for electrochemical sensing because the molecules are electrochemically inert within the aqueous potential window. When the electrode itself is fabricated on paper, that challenge is compounded by the need to integrate both recognition chemistry and reliable signal transduction onto a porous cellulose scaffold. The earliest proof-of-concept comes from Chi et al., developed a paper-based sensor using molecularly imprinted polyaniline (MIP-PANI) for selective PFOS detection. The conductive PANI matrix enabled resistive sensing, showing a low detection limit of 1.02 ppt and a linear range of 1-100 ppt. Binding-induced changes in resistance were attributed to specific PFOS-MIP interactions, confirmed by XPS and ATR-FTIR. The system combines high sensitivity and selectivity with low-cost, disposable architecture. However, further validation in real samples and assessment of sensor stability remain necessary for practical deployment (Figure 4C).^62^

Adaryan et al. (2025) pushed the concept further by integrating the paper electrode into an organic electrochemical transistor (OECT) architecture. In their design, a Whatman filter-paper gate is coated with a PFOA-templated polyaniline MIP. The MIP-functionalized gate electrode enabled specific binding of PFOA, which modulated the ionic flux and led to measurable shifts in the drain current. The sensor demonstrated a detection limit as low as 0.4 ppt, with a linear response range from 1 ppt to 1 ppb, showcasing excellent sensitivity. The response time was under 2 min, and the selectivity was validated by minimal interference from other PFAS analogues, such as PFOS. The transistor-based architecture allowed for inherent signal amplification, operating at low voltages suitable for portable applications. Overall, this OECT-MIP system offers a highly sensitive, selective, and field-deployable electrochemical platform for real-time monitoring of PFOA in environmental samples (Figure 4D).^28^

These approaches demonstrate the growing potential of electrochemical platforms particularly those combining molecular recognition with conductive interfaces for ultra-trace, selective PFAS detection. Their integration into low-cost, scalable formats highlights a promising path toward decentralized, real-time environmental monitoring solutions.

### Industrial Micropollutants

Hydrazine is a category-1 carcinogen widely used in propulsion, polymerization and corrosion-inhibition processes, is a prime target for such technology because allowable concentrations in surface waters lie well within the detection capabilities of modern nanomaterial-modified electrodes. The most comprehensive demonstration of a true cellulose-based PAD for hydrazine is provided by Singh et al. Screen-printed carbon electrodes were patterned directly onto filter paper and modified with a CuO/reduced graphene oxide (rGO) composite, yielding a disposable three-electrode strip that operates by differential pulse voltammetry. The device exhibits a linear response from 1 to 5000 μM with a LOD of 0.795 μM, maintains good selectivity against common co-contaminants, and crucially was validated in spiked lake-water samples with satisfactory recoveries, demonstrating genuine field relevance.^101^ The study sets an important benchmark by integrating inexpensive cellulose microfluidics with catalytic nanomaterials while still achieving sensitivity compatible with environmental guidelines. A closely related but methodologically distinct approach was reported earlier by Mohan et al., who refer to a “droplet paper-based” platform in which CuO clusters are electrodeposited onto flexible graphite sheet (“graphite paper”).^102^ Although the substrate is not classical cellulose, this quasi-paper device miniaturizes a self-contained three-electrode sensor, reaching an LOD of 0.348 μM within a narrow 1-7 μM range at neutral pH.

### Heavy Metals

Heavy-metal ions such as Pb­(II), Cd­(II), Hg­(II) and As­(III/V) are now classified as “emerging pollutants” because even trace quantities pose chronic ecological and health risks, yet they increasingly appear in distributed sources that are not routinely monitored. Paper-based electrochemical devices offer an attractive route to on-site quantitation: cellulose provides a built-in microfluidic matrix for passive sample handling, while printed or laser-written electrodes enable low-cost, disposable stripping-voltammetric assays.

Hybrid polyester–paper strips have set a recent benchmark for field-ready performance. Raucci et al. incorporated a screen-printed carbon electrode and in situ bismuth film onto Whatman No. 1 paper laminated to a polyester support, achieving LOD of 0.5 ppb for Pb­(II) and 0.3 ppb for Cd­(II) after passive pre-concentration through the porous paper; the same strip delivered recoveries of 93-105% in drinking water, mussel extracts and human serum, underscoring both versatility and robustness.^103^ Sánchez-Calvo et al. developed a paper-based electrochemical sensor using carbon electrodes modified with either mercury or bismuth films for trace heavy metal detection. Both modifications allowed simultaneous quantification of Cd­(II), Pb­(II), and In­(III), while Cu­(II) was only detectable with mercury. Mercury-modified electrodes provided superior sensitivity, with detection limits of 0.4 μg/mL for Cd­(II), 0.1 μg/mL for Pb­(II), 0.04 μg/mL for In­(III), and 0.2 μg/mL for Cu­(II), across linear ranges of 0.1–10 μg/mL. Although less sensitive, bismuth films offered a greener alternative. The sensor demonstrated accurate metal quantification in tap water using the standard addition method, highlighting its effectiveness as a low-cost, disposable solution for real-world water monitoring.^104^

Bui et al. developed a PES which detects nitrate and Hg­(II) simultaneously in water samples. The carbon paper electrodes received two modifications through selenium particles for Hg preconcentration and gold nanoparticles for nitrate reduction catalysis and Hg stripping site creation. The device used differential pulse voltammetry (DPV) for nitrate detection and anodic stripping voltammetry for Hg detection through the same electrodes. The device reached detection limits of 8.6 μM for nitrate and 1.0 ppb for Hg which met U.S. EPA standards and successfully tested spiked lake water samples for on-site environmental monitoring.^105^ For arsenic (As), two complementary paper-based approaches highlight the need for Au surfaces and speciation control. Núñez-Bajo et al. electrogenerated Au nanoparticles directly on porous carbon paper working electrodes and used chronoamperometric stripping to quantify As­(III) with an LOD of 2.2 μg L^–1^ (2.4 μg L^–1^ for total inorganic As after KI reduction of As (V) in white wines. The paper electrode itself serves as both the microfluidic substrate and the transducer, enabling on-paper preconcentration and low-potential stripping of As(0).^106^ An et al. (2022) reported a paper-based platform that performs on-site chromium speciation by coupling a patterned microchannel to an integrated three-electrode cell. In this PAD, chromium (Cr­(VI)) is quantified electrochemically, while total chromium is measured colorimetrically after on-chip oxidation with Ce­(IV). The device delivered limits of detection of 0.01 mg/L for Cr­(VI) and 0.06 mg/L for total Cr, with linear ranges of 0.05-3.0 mg/L and 0.2-3.0 mg/L, respectively. It showed good precision (RSD < 9.2%) and recoveries between 93.5% and 106% in real water samples, confirming reliable field-ready speciation.^107^

Device architecture itself is now treated as a performance lever. The multifolding vertical-flow electrochemical paper-based devices (ePADs) of Soulis and Economou integrates five stacked pre-concentration pads; by simply folding the device, sample aliquots wick serially to a Bi-citrate-modified working electrode, delivering ≈ 6-fold signal amplification and simultaneous determination of Zn­(II), Cd­(II) and Pb­(II) in a single readout.^108^ Such passive concentration without pumps or external reagents is especially attractive for decentralized monitoring.

Materials nano-engineering has pushed detection limits even lower. Scandurra and Mirabella deposited Bi nanoparticles inside a Nafion film cast on conductive graphene paper, yielding an all-paper electrode with 0.1 ppb LOD for both Pb­(II) and Cd­(II) and stable response for at least three months.^109^ Simplicity and scalability remain parallel design drivers. Tasić et al. converted ordinary cardboard into a graphene-like electrode in a single CO_2_-laser pass; the resulting laser-pyrolyzed paper electrode reached an LOD of 6 ppb for Pb­(II) without any surface modification, illustrating a one-step, reagent-free route for rapid fabrication.^110^ Madzivhandila et al. used wax–screen printing in an origami format to introduce vertical and horizontal flows that filter debris from “dirty” waters prior to sensing, achieving ppb-level Pb/Cd detection in buffer and demonstrating the practicality of foldable 3-D paper architectures.^63^

The development of PES devices enables heavy-metal detection to transition from laboratory settings to outdoor field applications. The combination of cellulose as a capillary pump and passive preconcentrator enables heavy-metal detection at sub-ppb levels for Pb­(II) and Cd­(II) using printed/laser-induced/graphene-paper electrodes with bismuth nanostructures. Origami designs enhance detection signals while making the devices easier to handle and polyester-paper combinations provide improved durability and smartphone-based optical detection methods enable quantitative measurements. The development of field-deployable solutions for Hg and As detection requires the integration of on-paper pretreatment methods with user-friendly digital interfaces to achieve complete on-site monitoring capabilities. Table 2 represent ePADs across pollutant classes, summarizing analytes, fabrication, recognition elements, electrochemical methods, performance, and real-matrix validation.

**Table 2:** Paper-Based Electrochemical Sensors Serve as a Representative Platform for Detecting Various Pollutants Including Pesticides, Heavy Metals, Pharmaceuticals, and Endocrine-Disrupting Biomarkers^a^

Pollutant class	Analyte(s)	Substrate/fabrication	Recognition element	Electrochemical technique	LOD	Linear range	Response time	Matrix validated	Ref
Pesticides (OP)	OPs (aggregate; field soil/produce context)	Office paper; printed conductive strips; on-paper Prussian Blue + carbon black	BChE inhibition	NR (PB typically enables low-potential amperometry; inferred)	1.3 ng mL–1	Up to ≈3 μg mL^–1^ (full LR NR)	NR	Soil and fruit vegetables (in situ)	^24^
Pesticides (OP)	Chlorpyrifos	Screen-printed carbon on paper (EμPAD) with 100 μL hydrophobic well; Basolite Z1200 MOF	Acetylcholinesterase (AChE) inhibition	EIS	3 ng L–1	10–1000 ng L–1	∼5 s	Real samples reported (details NR)	^30^
Pesticides (OP)	Paraoxon (model OP)	Bacterial cellulose (BC) nanopaper (wax-printed + screen-printed SPEs modified with CB/PB nanoparticles); TEMPO-oxidized nanopaper for optical	Butyrylcholinesterase (BChE) + substrates (butyrylthiocholine for EC, indoxyl acetate for optical)	Chronoamperometry (0.3 V vs Ag/AgCl); Dual detection: electrochemical (thiocholine) & optical (fluorescence from indoxyl)	∼12 ppb	Electrochemical: 20–50 ppb; Optical: 20–100 ppb	Electrochemical: 2 min (reaction) + 10 min inhibition; Optical: 5 min (reaction) + 20 min inhibition	Wastewater (paint industry effluent), spiked at 30–50 ppb, recovery 98-107%	^29^
Pesticide (Fungicide, Carbamate group)	Carbendazim	Screen-printed carbon electrodes (SPCEs) on kraft paper (SPCE/K) and parchment paper (SPCE/P); optimized by electrochemical activation in acidic medium (SPCE/K-a)	Non-enzymatic (direct electro-oxidation of carbendazim)	DPV	0.06 μM	0.5–10 μM	Few minutes (DPV scan + sample prep <10 min)	Apple skin and cabbage skin (non-destructive, in-situ monitoring); recoveries 96-104%	^111^
Heavy metals	Cd(II), Pb(II), Zn(II)	Multifolding vertical-flow ePADs with 5 preconcentration layers; hydrophobic barriers by pen-plotting; electrodes screen-printed; WE bulk-modified with bismuth citrate	Non-enzymatic; in situ generated Bi nanoparticles for alloying during preconcentration	Square wave anodic stripping voltammetry (SWASV); also tested DPASV and CCSA	Cd: 0.9 μg L^–1^; Pb: 0.6 μg L^–1^; Zn: 2.7 μg L^–1^	5–35 μg L^–1^ (for each metal)	Deposition time optimized at 240 s; full assay ∼10 min	Phosphate fertilizer (Cd: 10.6 ± 1.3 μg g^–1^; Pb: 4.1 ±0.6 μg g^–1^; Zn: 88 ± 9 μg g^–1^); Honey (spiked at 0.1 mg kg^–1^, recoveries 97–105%)	^112^
Heavy metals	Cd(II), Pb(II)	Whatman No.1 chromatography paper with paraffin screen-printed hydrophobic barriers; three-electrode system (carbon paste WE/CE, Ag/AgCl RE); gold nanoparticles + seed solution amplification	Aptamers for Cd(II) and Pb(II), labeled with ferrocene (Cd) and methylene blue (Pb)	Square wave voltammetry (SWV)	Cd: 23.31 pmol L^–1^; Pb: 46.23 pmol L^–1^	0.1–1000 nmol L^–1^ (for both Cd and Pb)	15 min (total detection)	Orange and lettuce (spiked at 5–50 nmol L^–1^); recoveries 93.2–95.8%	^113^
Heavy metals	Pb(II), Cd(II)	Graffoil sheet electrode drop-cast with ZIF-67/rGO composite; optimized deposition potential and time	Non-enzymatic (adsorption via imidazole groups in ZIF + oxygenated rGO sites)	Square Wave Anodic Stripping Voltammetry (SWASV)	Pb: 5 ppb; Cd: 2.93 ppb	5–50 ppb (linear) and 60–100 ppb (linear)	Deposition 350–400 s + stripping (∼10 min total)	Aqueous solutions (lab-prepared); high selectivity shown against Ni, Se, As, Zn interference	^114^
Pharmaceuticals/Forensic drugs	Lidocaine (LID)	Whatman chromatography paper treated with sodium tetraborate (fire retardant), coated with Ecoflex, then patterned by CO_2_ laser-scribing to form graphene electrodes; WE modified with MWCNT/PEDOT:PSS nanocomposite	Non-enzymatic (direct electro-oxidation of LID)	Differential Pulse Voltammetry (DPV); also characterized by CV and EIS	0.72 μmol L^–1^	31–248 μmol L^–1^	∼15 s per measurement (portable, in-field)	Spiked vodka and tequila (recoveries 78–119%); injectable anesthetic (20.1 ± 2.6 mg mL^–1^, matched label); seized cocaine samples (screening, qualitative detection)	^115^
Pharmaceuticals (Analgesic/antipyretic drug)	Paracetamol	Fully printed paper-based 2D device: Whatman No.1 chromatography paper with wax-printed hydrophobic channels; integrated screen-printed electrodes (carbon + Ag/AgCl)	Non-enzymatic (direct electro-oxidation of paracetamol)	Differential Pulse Voltammetry (DPV)	0.7 μM (standard solution); ∼1.3–1.5 μM (wastewater)	0–50 μM (tested range)	1 min (flow + mixing) + DPV scan (∼seconds)	Incoming and outgoing wastewaters from treatment plants (recoveries 82–98%)	^116^
Endocrine-disrupting/Stress hormone biomarker	Cortisol	Fully inkjet-printed Au electrodes on Whatman cellulose paper; integrated WE/RE/CE; functionalized with CMA via diazonium chemistry	Anti-cortisol monoclonal antibody (immobilized with EDC/NHS, blocked with ethanolamine)	Electrochemical Impedance Spectroscopy (EIS)	1.18 ng/mL (standard solution), 1.09 ng/mL (artificial saliva), 0.81 ng/mL (human saliva)	5–20 ng/mL (physiological range)	∼30 min incubation +5 min EIS	Human saliva (spiked samples); Artificial saliva; Standard solutions	^117^

a The table presents a summary of analytes together with paper substrates, fabrication methods, recognition elements, electrochemical techniques, analytical performance data (LOD, linear range, response time) and validation matrices from existing literature.

The promising sensitivity of ePADs remains limited by several challenges which include enzyme and affinity format cross-reactivity leading to false positives, device instability, signal drift and poor performance in complex matrices and insufficient long-term stability data and no inter-laboratory validation which prevent regulatory approval.

## Overcoming Barriers to Field Deployment: Technical, Digital, and Regulatory Pathways

Academic development has been fast but there is no evidence that ePADs for environmental monitoring have reached commercial deployment at scale by September 2025. The commercialization stage of ePAD remains in its initial development phase because laboratory prototypes must first progress to productized test stages to become market ready. In contrast, related technologies that use disposable screen printed sensor cartridges on plastic or ceramic substrates have already reached the market, for example the Palintest Kemio platform for disinfectants and heavy metals.^118^ The industrialization of electrochemical cartridges is still possible but the need for paper substrates hinders the advancement of this technology. The study needs to analyze all possible barriers which prevent ePAD from being implemented.

The deployment of paper-based electrochemical sensors faces multiple obstacles which unite into three main categories including technical issues, digital and operational aspects and regulatory and compliance requirements. Scientists encounter laboratory solutions that they control in their work but environmental samples never match these controlled laboratory solutions exactly. The sensor output becomes unstable when pH levels, temperature conditions, ionic strength and organic load concentrations change. The mixture of unfiltered water with soil slurries and food extracts produces an environment which makes cross reactivity and signal drift more probable. The research lacks standardized benchmarking protocols which represents another study limitation. The reported figures of merit that include limit of detection and linear range and recovery values are usually determined under particular conditions which create challenges for regulatory assessment and limit the ability to compare results. The majority of platforms function best for detecting individual analytes but field programs need to analyze multiple contaminants at once. The market requires integrated on paper pretreatment modules and multi sensing arrays. The absence of standardized benchmarking protocols hampers comparability of results, and this naturally links to a deeper issue: the limitations of paper substrates themselves, which simultaneously enable and constrain sensor performance.

Paper as a three-dimensional, porous reagent-reservoir and flow path solves many portability problems, but it also introduces mass-transport and stability penalties that are chronic in field work. The origami ePADs solve major problems that exist in field applications for heavy metal detection. The multi-fold design of Soulis et al. achieves sub-microgram sensitivity through pre-concentration layers while maintaining portability and disposability.^108^ The version developed by Madzivhandila et al. provides improved durability through its flow guidance system and particulate filtering mechanism and it can be manufactured at scale using printed methods.^63^ The research demonstrates how smart folding techniques enhance sensor performance which brings paper sensors nearer to practical water monitoring applications. Nanostructured inks are now routine for boosting electroactive area and lowering limits of detection into the low-nM or μg L^–1^ regime.^119–121^ Where the record thins is long-term durability: only the integrated array work of Alam et al. tracks sensor response for >24 h under continuous flow.^122^ Almost no publication supplies week-to-month data on calibration drift, fouling recovery, or shelf-life under cyclic humidity/temperature metrics that would be mandatory for regulatory acceptance. The origami-based solutions demonstrate potential to connect laboratory designs to field applications but their short operational time remains a major obstacle because they require digital and operational systems to conduct real-world monitoring.

Digital and operational barriers require attention. Platforms must deliver reliable data streams that integrate with existing environmental information systems. Without robust data pipelines, cloud based analytics, and interoperable formats, even technically strong devices may be underused. Signal processing, calibration algorithms, and intuitive user interfaces are necessary to support non expert operators in the field. Only one integrated system study couples PES-like electrochemical arrays with low-power electronics, on-board signal processing, Bluetooth telemetry and a smartphone app; even there, cybersecurity (encryption, firmware signing, audit trails) and long-term power autonomy are not rigorously analysed.^122^ Most reviews reference potentiostat miniaturisation or “IoT connectivity” as future necessities.^120,121^ Furthermore, machine learning algorithms can now be embedded into portable devices for pattern recognition, baseline correction, and automated anomaly detection, enhancing the robustness of field data.^123,124^ However, challenges remain in achieving seamless miniaturization, energy efficiency, and rugged design for harsh environments. Currently, there is no standardized ecosystem that unifies sensor data streams into scalable, secure, and regulatory-compliant monitoring platforms. The absence of end-to-end pipelines for sensor-to-cloud data acquisition and processing significantly restricts the broader implementation of PES networks, especially in decentralized or low-resource settings. The absence of standardized digital ecosystems stops all technical devices from achieving their maximum capabilities even when they have advanced technology. The path to data readiness faces a challenge because it needs regulatory systems to achieve official approval.

Regulatory and compliance hurdles form a third barrier. The lack of harmonized performance standards prevents consistent evaluation, while limited evidence on long term stability and inter laboratory validation constrains acceptance as official monitoring tools. Active collaboration with standard setting bodies, coupled with demonstration of reproducibility and reliability in real world trials, will be essential to build confidence and enable certification. Although electrochemical techniques are established in clinical and industrial settings, their disposable, paper-based counterparts lack formal validation protocols or standardized approval routes. The U.S. EPA and European Chemicals Agency require regulatory bodies to demonstrate reproducible long-term performance in complex matrices. The existing literature shows that PES systems have failed to meet these standards only occasionally.^125^ Moreover, the reliance on spiked buffer tests, rather than authentic environmental samples, undermines confidence in field applicability. The study by Platero et al. represents one of the few genuine field demonstrations that have been conducted for lead and arsenic monitoring on the Navajo Nation. These deployments usually last from a few hours to days and focus mainly on classical metals, which does not provide much information about long-term drift or fouling behavior when applied to pharmaceuticals, PFAS, or pesticides.^121^ Institutional adoption is hampered by factors that go beyond analytical figures of merit. Reviews point out that laboratories lack standardized training modules for non-specialist operators, and that existing quality-management systems provide no templates for integrating disposable, single-use sensors or for ensuring legally defensible data chains; these gaps foster procurement inertia and doubts about traceability.^121,126^ The built-in sustainability of paper-based electrochemical sensors makes them suitable for worldwide use but regulatory challenges must be overcome to achieve institutional adoption which will drive progress toward SDG achievement.

Nevertheless, PES technology aligns well with broader sustainability and public health goals. Its low-cost, biodegradable, and low-power profile makes it suitable for decentralized monitoring, particularly in low- and middle-income countries (LMICs). This aligns with United Nations Sustainable Development Goals (SDGs), especially SDG 6 (Clean Water and Sanitation) and SDG 12 (Responsible Consumption and Production).^127^ In summary, overcoming these barriers will require more than technical refinement. Standardized benchmarking protocols, dependable digital infrastructure, and proactive regulatory engagement are all critical to move paper based electrochemical sensing from the laboratory into scalable and durable field deployment. Figure 5 highlights the principal obstacles to this transition and the pathways needed for resolution.

**Figure 5:**
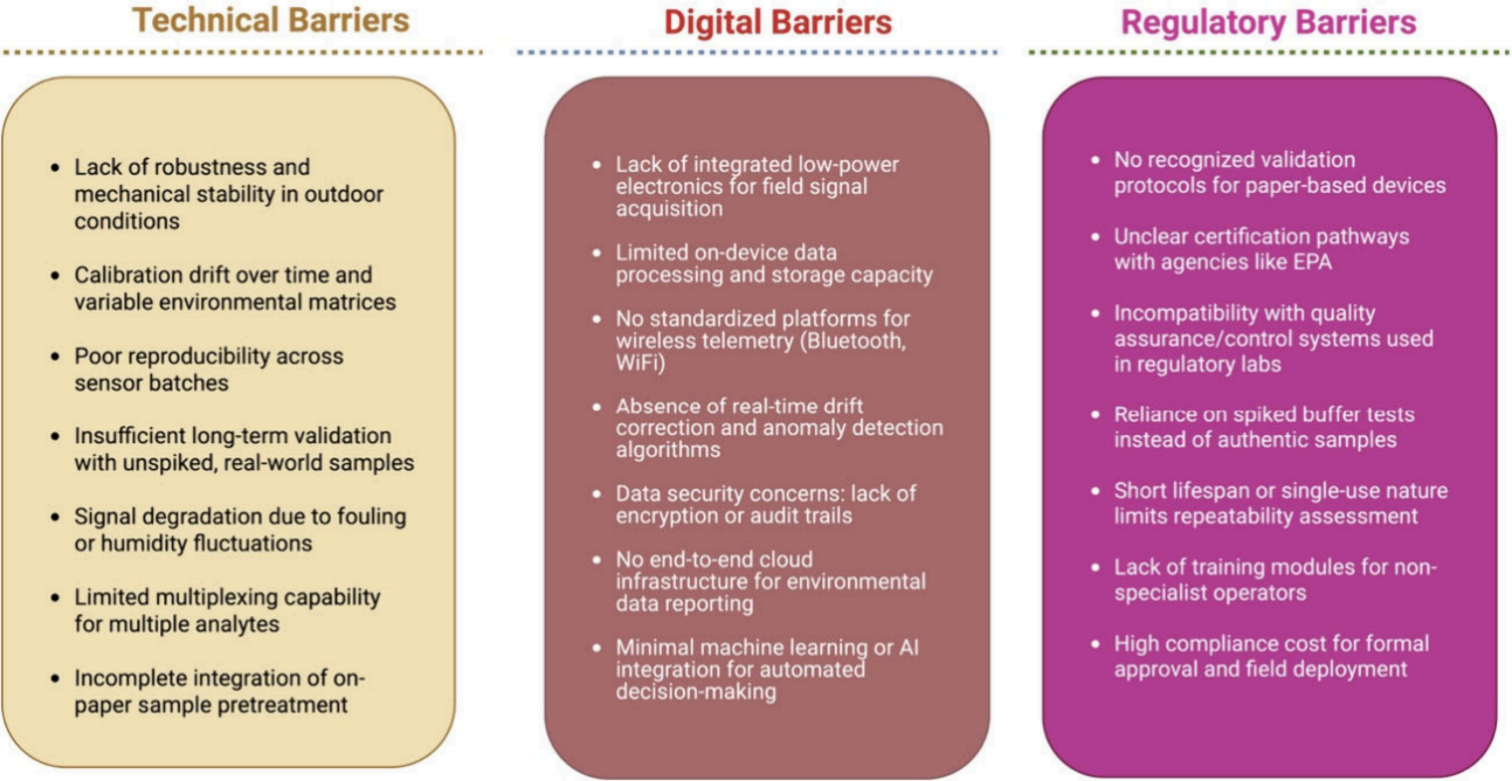
Barriers to scaling paper-based electrochemical sensors: technical (robustness, drift, reproducibility, longevity, limited multiplexing/pretreatment), digital (power/storage, nonstandard telemetry, weak drift correction/security, low cloud/AI use), and regulatory (no harmonized validation, unclear certification/QC fit, reliance on spiked buffers, short lifetimes/training gaps, high compliance cost).

## Future Outlook

Paper has matured from a passive substrate to an active analytical micro-factory capable of housing every step of environmental diagnostics from sample handling and analyte pre-treatment to signal transduction and data transmission. The unique foldability, porosity, and chemical versatility of cellulose make it an ideal foundation for the next generation of PES. These traits align seamlessly with the principles of green analytical chemistry, and they support the development of fully decentralized, reagent-free sensing platforms for diverse environmental matrices without the need for external pumps or laboratory infrastructure.^128^

A pivotal frontier in advancing PES lies in the integration of on-paper sample pre-treatment modules. True multi-analyte sensing in complex samples typically requires sequential operations such as pre-concentration, matrix cleanup, pH adjustment, or derivatization before detection can occur. Architectural innovations such as origami-folded and 3D-stacked microfluidic paper devices offer a blueprint for embedding such steps directly into the cellulose matrix. These designs create compartmentalized flow paths and functional zones, enabling multi-fold enrichment and chemical conditioning prior to electrochemical readout. As such, future PES platforms will not merely be sensing devices, but complete lab-on-paper systems.^129^

Equally transformative is the convergence of PES with digital sensing ecosystems. Smartphone-based potentiostats, Bluetooth modules, and Arduino-controlled interfaces have already begun to digitize electrochemical signal acquisition, allowing for real-time field analysis by non-specialists.^30^ As this trend evolves, the incorporation of artificial intelligence for signal processing, and cloud-based platforms for geotagged data aggregation and visualization, will unlock new possibilities for high-resolution spatial mapping of pollution events. Such systems could enable citizen scientists, farmers, and municipal workers to contribute meaningfully to decentralized environmental surveillance networks.

From a sustainability perspective, the field is undergoing a profound shift from rhetorical commitments to quantified eco-design. Recent work by Kumar and Maiti (2024) and others stresses the importance of integrating life cycle assessments (LCA) into sensor design.^130^ This includes selecting biodegradable substrates, adopting solvent-free and energy-efficient fabrication methods, and eliminating single-use plastics in favor of cellulose-based alternatives. These design strategies not only reduce material and energy burdens but also align directly with Sustainable Development Goals-particularly SDG 12 (Responsible Consumption and Production) and SDG 13 (Climate Action).

Yet, field validation and regulatory harmonization remain critical challenges. Despite the growing sophistication of PES technologies, most systems remain untested against internationally recognized protocols such as those issued by the U.S. EPA or WHO. Additionally, there is a shortage of long-term studies demonstrating reproducibility and reliability in unspiked, real-world environmental matrices. Moving forward, rigorous field trials across diverse ecological zones particularly in resource-limited settings which are necessary to establish trust, reproducibility, and policy relevance. Collaboration with regulatory bodies will be essential to formalize certification pathways and performance standards, ensuring that PES technologies can be adopted within existing legal frameworks for water, soil, and food safety monitoring.

In summary, the future of paper-based electrochemistry lies in its potential to merge analytical rigor with environmental sustainability, technological simplicity with digital sophistication, and decentralized access with regulatory reliability. Through multidisciplinary innovation and cross-sector collaboration, PES can evolve into next-generation diagnostic tools that are not only cost-effective and scalable but also capable of supporting global efforts toward environmental justice and sustainable development.
